# On Chancre of the Meatus and Urethra in the Male

**Published:** 1880-08

**Authors:** James Nevins Hyde

**Affiliations:** Professor of Skin and Venereal Diseases, Rush Medical College, Chicago


CHICAGO MEDICAL

JOURNAL & EXAMINER.

Vol. XLI.—AUGUST, 1880.—No. 2.

### Original Communications.

Article I.

### On Chancre of the Meatus and Urethra in the Male.

By James Nevins Hyde, M.D., Professor of Skin and Venereal Diseases, Rush Medical College, Chicago.

The anatomical and physiological objections to the location of the initial lesion of syphilis fairly within the canal of the male urethra, are obvious. While, with some individuals, the external orifice of that canal is capable of admitting the extremity of the little finger, it is in the majority of men far less capacious. Whether, however, that orifice be above or below the average size, the canal itself is plainly in all cases planned only to convey fluids in one direction, viz., from within outward. The "set" of every follicle and duct is evidently opposed to a current in the reverse direction. When in a normal state, it is traversed by the outgoing urinary stream with far greater ease than it is penetrated by the most skillfully propelled fluid intended as an injection. Every surgeon knows that a filiform bougie which refuses to enter a tight stricture approached by the face, when entered from behind through a fistulous sinus will often slip through the coarctation, from behind forward, with perfect readiness, occasionally without the knowledge of the operator. The urethra, as Sir Henry Thompson has well shown, is not a tube, but a long closed valve, open only when distended by natural or artificial measures. That this valve should open naturally to receive infectious secretions and thus become the seat of chancre, seems in the highest degreee improbable.

This a priori improbability, founded upon the nature and office of the valve, can not be safely ignored in the study of doubtful cases. The laws by which the body is regulated in health, are really formulated by ourselves from averages of observation, and the laws established regarding disease, are in the same measure genuine and trustworthy. The exceptions may be, according to their character, rare, curious, valuable, interesting or wholly unimportant.

Now since the day when Hunter's error was replaced with a clear recognition of the distinction between syphilis and gonorrhœa, few skillful and conscientious physicians have treated a genital discharge, incurred during suspicious intercourse, without a mental query as to the possibility of the existence of a lesion of syphilis in the urethra. That vulgar dishonesty or ignorance, which, confusing the symptoms of the two disorders, serves only to alarm the patient affected with clap by picturing to him its remote injurious influences upon the system at large, need not be here considered. In how many instances has the really conscientious practitioner, carefully inspecting the purulent discharge from the urethral orifice, and the small portion of the membrane of the canal which can be exposed by a separation of the labia, hesitated before answering the inevitable question of the patient, "Doctor, has this any connection with syphilis?"

The interest attaching to this subject seems to me to belong chiefly to those primary lesions of syphilis, which lie entirely within the urethral orifice, and which are therefore either invisible without the aid of endoscopy, or recognized with difficulty and imperfectly by the unaided eye. Under the title, "urethral chancre," or, "chancre of the urethra," several writers have included, not merely the lesions just mentioned, but those also located upon or near the urethral orifice, involving in whole or part, the rim of the canal, characterized by induration, discharge, and, occasionally by orificial stenosis after cicatrization. But in this last-mentioned class of cases, the syphilitic sore is evident, easily recognized by the naked eye, and accompanied by symptoms which only great inexperience or gross carelessness could attribute to a blennorrhagic affection.

From this failure to distinguish between the two varieties as to location described above, has arisen a misapprehension regarding the frequency of occurrence of the lesion. "Chancres of the urethra," say Bumstead and Taylor in their recently published treatise,* "are more frequent than is commonly supposed, and much more common than the chancroid in this locality." This statement is accurate in the sense in which the term, "chancres of the urethra" is employed by our authors, for they include under the designation, chancres of the meatus. In any other sense, the statement is far from being true. Of those lesions which are strictly intra-urethral, either invisible or but very imperfectly visible to the eye unaided by the endoscope, it can be properly said that they are of much less frequent occurence than is commonly supposed. These are they which awaken the anxiety of the physician and the alarm of the patient, in many cases where the diagnosis of gonorrhœa is attended with some doubt. I shall attempt to relieve the one and the other in the pages which follow.

During the last eighteen years, I have had the opportunity of observing, both in private and hospital practice, several thousand cases of primary syphilis, and of the entire number but two have been recognized as unquestioned instances of an initial sclerosis situated entirely within the urethral canal. In his valuable and compendious handbook of venereal disease,† my friend, Dr. Keyes, states that only two similar cases have been observed by him, one of these through the endoscope.

Mr. Henry Lee remarks that "an indurated sore very rarely exists within the urethra although the syphilitic poison must be often conveyed there. On the lips of the urethra it not

* The Pathology and Treatment of Venereal Diseases, Phila. 1879, p. 471.

† New York, 1880, p. 89.

unfrequently occurs, but in the whole course of my experience I have never known it to originate further back than a quarter of an inch from the orifice: and in the great majority of cases, if it affects the urethra at all, it spreads to it from without." (Lectures on Syphilis, etc., Phil., 1875, p. 63.) A brief record of the essential features in my cases is given below.

Case 1. B—, a florid gentleman, 50 years of age, and well nourished, supporting both wife and mistress, had consulted me frequently for progenital herpes. On the 15th of Aug. 1879, five days after intercourse with his mistress, he presented himself with a circlet of herpetic vesicles surmounting the corona glandis, having the appearances noted in his previous attacks. They disappeared as usual after the part was properly protected and washed for a few days with an astringent and sedative lotion. On the first of the next month, he returned for examination, and at this time his vesicles were all healed. But I found an indurated gland in the right groin, and a pleiad similarly involved on the other side. There was no induration of any portion of the mucous membrane lining the preputial sac, and it presented a normal appearance, as did also the membrane of the canal which was visible when the meatus urinarius was exposed by separating the labia. There was, however, a slight, very slight, discharge of mucoid matter from this orifice, and a mild degree of subjective discomfort.

On seizing the glans vertically between the thumb and the finger, and pressing gently upon it, from before backward while the organ was in the pendulous position, the sensation produced was suggestive of a short section of the stem of a common clay pipe set in the substance of the glans where it is tunneled by the urethra. This was evidently produced by a dense, resilient, well localized neoplasm, recognizable with perfect ease by the touch alone, and neither by the discharge which it occasioned nor by other concomitant symptoms, liable to be confounded with a gonorrhœal affection.

Endoscopic examination, made in the manner described below, revealed the mucous lining of the fossa navicularis in a thickened, and infiltrated condition, while a light-greyish-hued, secreting, linear abrasion, at the depth of one and one-half centimeters,

($\frac{3}{4}$ inch), itself about five millimeters in length ($\frac{1}{4}$ inch), and parallel to the axis of the canal, could be recognized with great distinctness in the right wall. When its secretion was carefully wiped away, the latter was seen to proceed from a superficial loss of substance on the right side of the canal, having the dimensions given above. It was clear that the induration lay beneath this erosion and that the latter was in intimate connection with it, extending beyond its borders in every direction. The color of the urethral membrane in no way suggested the intense redness of acute blennorraghia. On pressure there was evoked a very slight tenderness. In short the pronounced symptoms of the case were the induration, which was very clear to the touch without the aid of endoscopy, and the coincident adenopathy. Only the grossest carelessness could have served to misinterpret the meaning of these significant features.

The abrasion having been dressed with powdered iodoform applied on a wisp of cotton with a small probe, the indurated mass did not fail to disappear gradually, the patient meantime being brought under the influence of small doses of the protiodide of mercury. Within five weeks a typical macular syphiloderm appeared over the skin of the belly and chest, very slightly evident upon the face. This yielded promptly to fumigations with the vapor of calomel and cinnabar; and the subsequent history of the patient has been the common one, of crops of mucous patches appearing at intervals on the mucous membrane of the mouth and especially of the tongue, the latter influenced doubtless by the habits of the patient as regards tobacco.

Soon after the diagnosis in this case was established, I had the opportunity of "confrontation" with the mistress from whom the disease has been presumably acquired. She admitted to me, in private, unfaithfulness to her lover, and exhibited normal external genitalia. But pressure extruded a sanguineous and purulent discharge from the contracted os of a virgin uterus, which probably issued from a secondary lesion situated on the wall of the neck, as she had also post-cervical adenopathy, faucial hyperæmia with sequelæ of old ulceration, and two typical gummata over the left olecranon. She stated that the coitus, suspected to have been the occasion of the trouble, had been accomplished with considerable violence; that she had not been feeling well for two weeks prior to that date; and that the act had been followed by a slight bloody discharge from the vagina which both parties had noticed. Beneath the rouge of her artificially reddened cheeks, a distinct pallor existed. Her case was one of those not rarely seen in women, where the symptoms of the disease pass unnoticed for a variable period, and are excited to activity by various depressing causes. She was placed upon specific treatment and her symptoms were succeeded by those of fair health in the course of two months. The relation between the parties was ended by the discovery of the facts.

CASE II. G., æt. twenty-four, a thin, nervous and cachectic looking youth, came to me in February, 1880, by the advice of a colleague, for the relief of his symptoms. He had been leading a dissolute life, indulging with many women, and could give no details as to the length of time during which he had suffered from venereal disease, except that he thought it was "several weeks." On examination his skin was seen to be quite generally covered with a classical macular syphiloderm; there was post-cervical, and inguinal adenopathy, pustules of the hairy scalp, mucous patches of the mouth and lips, and moderate alopecia. When I examined for the seat of the primary lesion, I was surprised to find that the entire mucous surface of the glans was desquamating, though apart from the rolling off in minute flakes of the outer layer of epithelium, there was no evident lesion, nor trace of any such having previously existed. As soon, however, as the glans was seized with the thumb and fingers, in the manner described above, it became clear that a large mass of dense induration lay deeply imbedded within its substance. The dry, thickened and toughened feel of the entire extremity of the organ was peculiar, and its appearance suggested at first sight, the diphtheroid features which certain primary lesions of this part occasionally exhibit. The real character of the sclerosis was, however, easily determined when examined with a little care. The induration suggested the feeling of a No. 17 F. sound inserted in the urethra to the extent of a few centimeters and the consequent thickening was such that the lips of the urethra were stiffened by reason of loss of their natural elasticity, and were penetrated with some little difficulty by the tube of the endoscope. This once inserted, there became visible a peculiar bloodless, whitish, cicatriform condition of the lining membrane, possibly brought about in part by the pressure upon the infiltrated walls by the encircled tube. It was evident that, in this case also, the neoplasm was submucous. At one point in the roof, about two centimeters (three-fourths of an inch) from the urinary orifice, was a large-pin-head-sized, slightly elevated granulation, of much lighter color than the similar granulations commonly seen upon the free mucous surfaces of the external genitals. In this case, the discharge was so slight as to scarcely awaken the apprehension of the patient, who, it may be said, however, was one of those who exhibit a not unusual indifference to the diseased state of the external generative organs.

Here was clearly an instance of the so-called "transformation of chancre in situ," occurring in the urethra, the primary lesion having been transformed into one of the secondary stage, co-existing thus with the outbreak of a cutaneous exanthem and other manifestations of systemic trouble. The occurrence of the singular desquamation upon the glans, was explainable by supposing that a degree of inflammation had complicated the sclerosis, and that the extension of this latter to the covering of the glans had brought about the destruction of its most external epithelial layer.

The local and constitutional symptoms in this case yielded gradually though somewhat slowly to appropriate treatment, begun by the fumigations described above, and continued by the administration of the biniodide of mercury by the stomach. The only local treatment necessary was an inunction of the surface of the glans with carbolized oil, after washing with soap and water, and one or two pencilings of the vegetating urethral surface with a twenty per cent. solution of nitrate of silver. All three patients named above are still undergoing a gentle mercurial course, and all are free from symptoms of active manifestation of the diathesis with the single exception of the male patient in case No. 1, who is still annoyed at times by the occurrence of patches on the dorsum of the tongue.

It may be well to remark in passing, though the matter is not strictly germane to the subject under consideration, that upon repeated and apparently trivial attacks of preputial herpes, a guarded prognosis should always be pronounced in the case of individuals who admit that they have indulged in illegitimate sexual relations. Every one knows that an attack of herpes, pure and simple, is never followed by syphilis, but many are apt to forget that an herpetic lesion of the prepuce or glans, is an admirable portal for the infecting virus of both forms of contagious venereal sore. Men subject to this annoyance, leading irregular lives, are many times lulled into a false sense of security by discovering upon their persons a familiar local disorder, which up to date, has always proved benign in character, but which, after inoculation, or even when the vesicle is merely an expression of protest at the point where syphilitic inoculation has already occurred but not yet declared itself, does not fail afterward to exhibit a characteristic induration, and to be followed by the general symptoms of lues.

For the endoscopic examination of the pendulous portion of the male urethra, a simpler apparatus than the endoscope of Désormeaux has been used by me with advantage. It consists merely of the ordinary straight endoscopic tube, with a Miot lamp, by which illumination is secured. At times, it is well to directly examine the lining membrane through the aperture of an ophthalmoscopic mirror, the light being reflected from the Miot lamp or from a gas jet at the level of the eye, the patient being in the erect position before the operator. When for any reason, it is desirable to make examinations in the recumbent position, the Miot lamp can be held at any angle, and the rays directed so as to fall into the tube by the side of the head of him who inspects the canal.

An important point to note, in the examination of the canal of patients who are affected with chronic or subacute urethral discharges, is the frequent discovery upon the floor of the fossa, of something which looks at first sight, very like an ulcer. The surgeon who has not had experience in this matter, is exceedingly apt to mistake for an ulcer, syphilitic or simple, the appearance presented at this point. It is due merely to the fact that the eye is habituated to representations of the urethra after vertical section, and the post-mortem view of the floor of the canal, when the urethra is laid flat upon a plane surface, gives no clue to the tinted picture presented during life. In the large number of men who have even a slight urethral weeping, there seems to be a tendency of the secretion to accumulate in the little pocket on the floor of the fossa, which becomes visible when the endoscope is introduced at this point. Here a linear, slightly reddened, or yellowish longitudinal furrow is evident, covered with a more or less opaline scanty mucus, which can be removed by a very simple manipulation, when the supposititious "ulcer" is at once seen to be the normal reddish sulcus which, even in entirely healthy individuals, exhibits a color somewhat heightened above that of the urethral walls. The following case illustrates the point to which reference is here made:

CASE III. Dr. W., aet. forty-two, married, narrow-chested and poorly-nourished, with a history of incipient tuberculosis, contracted a urethral discharge in Paris, after coitus with a suspicious party. The discharge appeared within six days after exposure, and, being then in London, the patient consulted a British practitioner of repute, who pronounced the case one of simple gonorrhœa, and prescribed accordingly. Dr. W. then returned by transatlantic steamer to this country, and while on his homeward voyage, his clap improved perceptibly. Some slight gleety discharge however remaining, he consulted a professional gentleman of acknowledged skill in the city of New York, who at once proceeded to make an endoscopic examination of the urethra. The physician-patient was a man of intelligence, and was proportionately shocked to learn, from the lips of his colleague, that he was affected with a urethral chancre. The operator with the tube was not only so confident of the existence of this primary lesion of syphilis, but so charmed by its exceptionally pronounced features, that he expressed regret because he had not an arrangement of mirrors by which the patient might, auto-endoscopically, inspect for himself the evidences of his dreaded disease. This practitioner was as confident as he was conscientious in his convictions, and at once placed the patient upon a mercurial course, medicating the urethral lesion with iodoform in powder, and on wax bougies made expressly for the purpose.

In the course of two weeks, the patient came to this city and consulted me, giving a full history of his case, and exhibiting in his general facies and condition, the evidences of the mental trouble which his supposed disorder had occasioned, as well as the effect upon his tuberculous diathesis of the mercurial course to which he had been subjected.

I discovered upon examination, that he had all the symptoms of a simple gleet, without stricture, and on introducing the endoscopic tube, could discover absolutely no lesion nor induration save the little pseudo-ulcer, which can always be detected in similar cases, upon the floor of the fossa, as has been pointed out above. In no respect did this differ from the same furrow-like chink, as seen in a dozen patients examined immediately before. At this date too, there was no adenopathy of any part of the body, and the catarrhal symptoms from which the doctor suffered, were those to which he had been long accustomed before his recent escapade. With considerable hesitation, he consented to accept my verdict in his case, a verdict to which he has since acceded with very great heartiness, as his gleet completely disappeared under appropriate treatment, and his general health manifestly improved soon after his mercurial medication was discontinued. Four months have since passed, and he has yet to exhibit the first symptom of syphilis.

Every physician knows that the muco-cutaneous tissues at the orifices of the outlets of the body are more sensitive to the action of external irritants than those which are situated either more deeply or externally. Thus, the nitrate of silver will produce more disagreeable effects upon the angles of the mouth, when applied there, than either upon the buccal membrane or the skin of the lip. This sensitiveness is clearly to be recognized in the case of visible sores of the meatus of the urethra. They are readily irritated by the urine, a fact which does not seem to be at all significant of deeper urethral lesions, and this irritability is apt to result in phagedena, a complication which is well known to be more common in the case of the chancroid than in primary syphilitic lesions. The resulting loss of tissue, occurring as it does at the apex of the cone of the glans penis, produces a striking and permanent deformity, giving the body a peculiar square-shaped appearance, and often interfering with the smoothness of the urinary jet, as it is projected from the body in the act of micturition. I append a brief record of two of many cases of chancre of the meatus observed by myself, in illustration of the facts set forth above:

*Case IV.*—S., a Jewish youth, 19 years of age, and circumcised duly in the ritual of his race, consulted me in March of 1877, with a small-penny-sized, ragged looking ulcer, on the right half of the meatus, involving equally the urethral and penile surfaces. It was irregularly oval in shape, its base indurated, its floor covered with a pultaceous slough, whose removal showed beneath a granular and livid surface. It was exquisitely sensitive, aggravated by each discharge of urine, and was evidently serpiginous in the direction of the right lateral surface of the glans. It had existed for more than two weeks, and had been first observed about one month before examination, after exposure with a woman of the town. The inguinal glands of both sides were indurated and one on the right was quite tender. The young man had an exceedingly cachectic look, and was put upon steel internally, and a lotion containing the potassio-tartrate of iron locally, after which the sore went on to cicatrization, leaving a decidedly blunt glans, which looked as thought a thin slice of tissue had been removed from the extremity, at an angle of about thirty-five degrees from the axis of the cone on the right. General syphilis declared itself later, and was of a mild grade.

*Case V.*—B., a newly-married artizan, fairly well-nourished, 25 years of age, was brought to me for consultation by a professional colleague. About twelve days after coitus with a suspicious party, he had a sore develop on the glans at the urethral orifice, which became quite painful and soon spread in extent. It had existed for some weeks prior to the date of examination. I established the fact of a conical, crateriform ulcer, involving the entire rim of the meatus, but penetrating more deeply on the inferior surface. The base of the cone was nearly circular and at the plane of the meatus, its apex buried in the urethra to the depth of twelve millimeters (one-half inch). It was densely indurated and its floor covered with reddish granulations. Tenderness was quite marked, and he complained of pain on micturition. The inguinal glands were enlarged as also those of the crural region, and moderately indurated. There was little discharge from the urethra or from the surface of the sore. The patient had been careful to separate from his wife, in order to spare her the risk of infection. He was put upon small doses of the protiodide of mercury and his sore penciled with a nitrate of silver solution containing ten grains to the ounce. Under this treatment the irritability diminished and cicatrization occurred, with the glans looking as though it had lost a small anterior section transverse to the axis, presenting thus a squarish shape. The urinary orifice was completely destroyed, and admitted a No. 30 F. metallic sound with ease, although his physician had before entered only a No. 18 F. during an exploratory examination for suspected stricture at a previous date. The patient complained, when questioned on the subject, of the irregular distribution of his stream in the act of micturition, as he frequently soiled his clothing at these times. His physician informed me, subsequent to the date of my observation, when cicatrization had been accomplished, that general syphilis followed.

The points of interest worthy of special note in these last cases, are, first, that in both instances the sores were syphilitic. It is scarcely necessary to remark that extensive destruction of tissue is not uncommon after soft chancre. I have seen three-fourths of the entire organ, inclusive of the urethra, disappear after gangrene had occurred in a case of chancroid of the frenum. Second, I remark, in passing, that circumcision, while it is deservedly supposed to diminish the chances of venereal infection, does not furnish complete immunity. The experience of those who see many patients affected with venereal disease, always includes a certain proportion of individuals of the Israelitish race. I have had under observation, during the current month, four Jewish patients, one treated for abscess of the prostate, the result of acute prostatitis complicating gonorrhœa, one for secondary, and the others for ancient syphilis. Third, the error should not be committed of prognosticating grave syphilis from gravity of the initial lesion, or its complications. It seems to be at present accepted as true, that there is no relation between either the extent of induration, the length of the period of incubation, the occurrence of phagedena, or other concomitants of primary syphilis, and the subsequent systemic phenomena. This fact, opposed as it may appear to what would be anticipated by a knowledge of pathology in general, rests upon the best of clinical evidence ; and while it is perfectly true that grave syphilis may follow a severe initial sclerosis, it is equally true that in other cases there is no such sequence, and a man with a severe serpiginous primary sore may afterward exhibit symptoms of the most benign form of the disease.

Of a total of 1773 lesions of primary syphilis, collected by Jullien in his recently published and voluminous treatise on the venereal diseases,* but 17 were strictly intra-urethral in location, while 89 involved the meatus. The author remarks that this initial sclerosis is almost never encountered deeper than the anterior half or third of the penile region, and that when the meatus is involved, the sore, incessantly irritated by the urinary current, presents the irregular appearance of the simple chancre. Respecting the best manner in which it may be recognized when seated more deeply, his remarks are seen to be justified by the clinical facts I have given above : "In order to best discover the induration, the urethral orifice must be seized between the thumb and finger through its vertical diameter. Compressed transversely, the tissues offer a resistance which permits but an exceedingly imperfect appreciation of the special sensation imparted by the syphiloma. The plastic infiltration may be tardy of occurrence, as in the case of a patient of Gailleton, whose chancre displayed induration on the thirtieth day of ulceration, and at a time when it was ready to cicatrize. At this moment the sclerosis developed to such a degree that the extremity of the canal seemed to be made of wood and micturition was, during several days, rendered difficult by a real fibrinous retraction."

He proceeds further to point out the painful character of the sore of the meatus, when irritated by the urine, and its tendency to phagedena, with the characteristic alteration in the configura-

---

* Traité Pratique de Maladies Vénériennes, Paris, 1879, p. 581.

tion of the glans, which follows the last named accident. Speaking of both forms, he states that the secretion to which they give rise, is "of too little importance to attract attention," and relies for diagnostic ends upon the presence of a little nodose mass more or less sensible to the touch and painful on micturition, together with the accompanying inguinal adenopathy.

It is manifestly improper to draw conclusions from a limited number of cases, but when this limited number is a series of rare exceptions to rules established in the experience of a generation of men, the inferences they suggest should possess some interest. I think it may be fairly concluded that:

1. Chancres of the meatus and of the urethra are symptomatically distinct.

2. Chancres of the meatus are chiefly remarkable for their irritability, in consequence of the passage over them of the urinary current, their tendency to phagedena, and the characteristic deformity of the glans which they frequently occasion.

3. Intra-urethral chancre, as distinguished from visible chancre of the meatus and chancre involving in part only the rim of the urethral valve, is of exceedingly rare occurrence.

4. Intra-urethral chancre, invisible or but imperfectly visible to the eye unaided by the endoscope, can usually be distinguished as such in consequence of the mass of induration which lies immediately beneath the urethral mucous membrane at the site of the lesion, and is readily recognized by the touch through the external tissues of the male organ.

5. Intra-urethral chancres are accompanied by very scanty or insignificant discharge, and when the latter is present, it bears no proportion to the evident signs of disease connected with the sclerosis.

6. The reddened furrow or sulcus, covered with a thin secretion, visible in the floor of the fossa navicularis, especially in the subjects of gleet, should never be mistaken for intra-urethral chancre.

7. When a man exhibits a decidedly purulent urethral discharge, without coincident symptoms of unmistakable syphilis, he can be safely pronounced free from all danger of the last named disease, provided always the period of incubation of syphilitic chancre has, in his history, already lapsed.

8. Intra-urethral chancre need therefore never be mistaken for gonorrhœal disease, as the two affections are so distinct that a differential diagnosis can usually be satisfactorily established without the aid of the endoscope.

9. For many reasons the endoscope should be regarded as a valuable adjuvant in the diagnosis of urethral disorders, and recourse should be had to it in all doubtful cases.

For the benefit of those who are specially interested in the subject under consideration, I append a list of a few illustrations in color, of the urethral lesions described:

From "Die Syphilis der Haut und der Angrenzenden Schleimhäute;" M. Kaposi; Wien, 1873. I Lieferung, Tafel I, 3. Soft Sore of the Meatus: T. V., 2 and 3. Chancres of the Meatus in Jewish Children, Infected in the Rite of Circumcision.

Also from the reproduction of Ricord's "Illustations of Venereal Disease," Philadelphia, 1872.

Plate VI., Fig. 3. Grayish-yellow secretion covering symmetrical urethral ulcers.

Plate IX., Figs. 1 and 3. Extensive ulceration of membranous and prostatic portion of the urethra, involving also the neck of the bladder, said to be instances of primary syphilis, but more probably later manifestations.

Plate XI., 1. Chancre of meatus.

2. Small grayish-hued ulcer in the superior commissure of the meatus, visible after separtion of the labia.

